# Fracture blisters: predictors for time to definitive fixation in pilon fractures

**DOI:** 10.1007/s00590-023-03623-w

**Published:** 2023-06-29

**Authors:** Avinaash Korrapati, Canhnghi N. Ta, Brendon C. Mitchell, Pelle V. Wall, Pradyumna Gurusamy, Kathryn Dwight, Paul J. Girard, Alexandra K. Schwartz, William T. Kent

**Affiliations:** Department of Orthopaedic Surgery, University of California San Diego, 200 W Arbor Drive, MC 8894, San Diego, CA 92103 USA

**Keywords:** Pilon fractures, Fracture blisters, General trauma, Case control, External fixation, Open reduction, Internal fixation

## Abstract

**Purpose:**

Fracture blisters, a common soft-tissue complication of pilon fractures, are associated with post-operative wound infections, delays in definitive fixation, and alterations in surgical plan. The purpose of this study was to (1) identify the delay in surgery attributable to the presence fracture blisters and (2) investigate the relationship of fracture blisters to comorbidities and fracture severity.

**Methods:**

Patients with pilon fractures at an urban level 1 Trauma center from 2010 to 2021 were identified. The presence or absence of fracture blisters was noted, along with location. Demographic information, time from injury to external fixator placement, and time to definitive open reduction internal fixation (ORIF) were collected. Pilon fractures were classified according to AO/OTA guidelines using CT imaging and plain radiographs.

**Results:**

314 patients with pilon fractures were available for analysis, eighty (25%) of whom were found to have fracture blisters. Patients with fracture blisters had longer time to surgery compared to those without fracture blisters (14.2 days vs 7.9 days, *p* < 0.001). A greater proportion of patients with fracture blisters had AO/OTA 43C fracture patterns, compared with those without fracture blisters (71.3% vs 53.8%, *p* = 0.03). Fractures blisters were less likely to be localized over the posterior ankle (12%, *p* = 0.007).

**Conclusion:**

The presence of fracture blisters in pilon fractures are associated with significant delays in time to definitive fixation and higher energy fracture patterns. Fracture blisters are less commonly located over the posterior ankle which may support the implementation of a staged posterolateral approach when managing these injures.

## Introduction

Pilon fractures are severe intraarticular injuries to the distal tibia that are most commonly the result of a high energy axial load. Soft tissue trauma, often indicated by the development of fracture blisters, is associated with these high energy injuries, and predisposes patients to wound complications and infection [1]. Current literature reports wound healing complication rates in pilon fractures ranging from 9 to 18% [1–3]. Fracture blisters pose a complex challenge for orthopaedic surgeons. They typically occur in areas without a safe envelope of subcutaneous tissue, develop between 6 and 72 h after the inciting injury [4, 5], and can interfere with surgical approaches to the tibial plafond based on their location. Temporizing early external fixation (EF) is often implemented to allow for soft tissue recovery, while providing initial fracture stabilization and allowing for monitoring of the soft tissues [6].

The pathophysiology of fracture blister development is multifaceted. With significant soft tissue trauma, shearing of the dermal–epidermal junction may occur at the time of injury and lead to the formation of a potential space. The edema that develops during the inflammatory phase increases both the interstitial and filtration pressure, leading to fluid accumulation in the potential space [4]. The increased colloid pressure in the epidermal or subepidermal space further contributes to fluid transfer. These forces act in conjunction to drive fluid into the potential space to form the fracture blister. Previous studies have found that the development of fracture blisters is associated with microvascular disease, including hypertension, smoking, alcohol abuse, peripheral vascular disease, lymphatic obstruction, and diabetes [4, 5, 7].

Fracture blisters carry significant clinical relevance and can greatly influence patient outcomes. They have been associated with postoperative wound infections, delays in operative fixation, and changes in surgical planning [8]. Giordano et al. [9] in a study of 53 patients with fracture blisters, reported wound healing complications in 7 (13.2%) patients. Similarly, Strauss et al. reported lower extremity soft-tissue complications associated with fracture blisters in 5 of 45 (11.1%) patients [10]. Early definitive fixation in areas prone to fracture blisters can lead to severe soft tissue complications, even leading to loss of limb [4]. Therefore, the current standard of care is for delayed or staged fixation to minimize the risk of wound complications in the setting of fracture blisters [4, 6, 11]. No clear consensus on the optimal management of fracture blisters exists.

Few studies have evaluated the delay in definitive surgical fixation when fracture blisters are present. Strauss et al. sought to evaluate the impact of fracture blisters on time to ORIF and found the mean delay in definitive treatment for patients with pilon fractures with fracture blisters was 6.8 days, though this was a limited sample size of only 4 patients [10]. In this study, we sought to identify the delay in surgery attributable to the presence fracture blisters and localize the distribution of fracture blisters relative to the distal leg and ankle in pilon fractures. Secondary goals of our study included evaluating the relationship between fracture blisters and patient demographics, comorbidities, and fracture classification (AO/OTA). This study aims to provide further insight into the management of pilon fractures with associated fracture blisters and the influence these injuries have on surgical planning.

## Methods

After IRB approval, our institution’s electronic medical record was queried for patients treated for pilon fractures between January 2010 and March 2021 by 3 orthopaedic trauma surgeons. A waiver of informed consent was granted by our institutional review board for the conduction of this retrospective study. Patient charts were reviewed for time from injury to external fixator placement and time from injury to ORIF. The presence or absence of fracture blisters on the injured extremity were noted, along with fracture blister location on the ankle (anterior, posterior, lateral, medial). Patient demographic information including age and gender, injury details including laterality and open versus closed fracture, and comorbidities, including diabetes mellitus, smoking status, alcohol use, hypertension, peripheral vascular disease, and lymphedema or lymphatic disorder, were also recorded. Pilon fractures were classified according to AO/OTA guidelines using available CT imaging and plain radiographs. Fracture blisters were treated with mechanical unroofing and application of xeroform or silver sulfadiazine (Silvadene) with daily dressing changes per surgeon preference. Readiness for definitive surgery was determined based on clinical soft tissue appearance, skin-wrinkling, and fracture blister re-epithelization prior to definitive ORIF.

Two-sample T-tests were used to compare quantitative variables while Chi-square (with Yates correction) and Fisher exact tests were performed for categorical variables. Comparisons to expected proportions and distributions were performed using exact binomial and multinomial goodness of fit tests respectively. *P* values of post-hoc analyses were corrected using the Bonferroni correction method. Statistical analysis was performed using R Statistical Software (R Core Team, 2020). Statistical significance was defined as *p* < 0.05.

## Results

A total of 316 patients with pilon fractures were available for analysis, of which two were excluded due to lack of preoperative imaging available. Eighty patients (25%) were found to have associated fracture blisters. There were no significant differences in patient demographics or comorbidities between the fracture blister and non-fracture blister cohorts (Table 1).

**Table 1:** Patient demographics

Characteristic	Blisters (n = 80)	No blisters (n = 234)	*P* Value
Mean Age (SD), yr	41.6 (14.6)	39.3 (14.0)	0.22
Sex, n (%)			
Male	65 (81.2)	163 (69.7)	0.06
Female	15 (28.8)	71 (30.3)	
Laterality, n (%)			
Left	37 (46.2)	122 (52.1)	0.44
Right	43 (53.8)	112 (47.9)	
Open vs closed			
Open	58 (72.5)	161 (68.8)	0.63
Closed	22 (27.5)	73 (31.2)	
Comorbidities (%)			
Diabetes mellitus	8 (10.0)	21 (9.0)	0.96
Hypertension	16 (20.0)	42 (17.9)	0.81
Smoking	39 (48.8)	96 (41.0)	0.28
Alcohol	40 (50.0)	102 (43.6)	0.39
PVD	1 (1.2)	5 (2.1)	1.00

Patients who presented with fracture blisters were found to have a significantly increased delay in time to definitive fixation when compared to those without fracture blisters (14.2 ± 7.8 days vs 7.9 ± 7.6, *p* < 0.001) (Table 2).

**Table 2:** Time from injury to definitive fixation

Presence of fracture blisters	Mean (SD) (days)	*P* value
Blisters (n = 80)	14.2 (7.8)	> 0.001
No blisters (n = 236)	7.9 (7.6)	

There was a statistically significant difference in AO/OTA classification distribution between the fracture blister and non-fracture blister cohorts. Post-hoc analyses found a significantly higher proportion of 43C fractures among patients with fracture blisters (71.3% vs 53.8%, corrected *p* value = 0.03). Patients with no fracture blisters were found to have a higher proportion of 43B fractures (44.0% vs 26.3% corrected *p* value = 0.02) (Table 3).

**Table 3:** AO/OTA classification

Fracture classification*	Blisters (n = 80)	No blisters (n = 234)	*P* value†
43A (%)	2 (2.5)	5 (2.1)	1.00
43A1	0	3	
43A2	1	1	
43A3	1	1	
43B (%)	21 (26.3)	103 (44.0)	0.02
43B1	2	24	
43B2	6	33	
43B3	13	46	
43C (%)	57 (71.3)	126 (53.8)	0.03
43C1	0	8	
43C2	16	41	
43C3	41	77	

*P = 0.02, comparison of AO classification distribution between fracture blister and non-fracture blister cohorts, fisher exact test

†2-sample test for equality of proportions. *P*-value corrected via Bonferroni correction method

There was a statistically significant difference in the distribution of fracture blister location as shown in Table 4. On post-hoc analyses, there was a significantly higher proportion of medial ankle fracture blisters (*p* < 0.001) and lower proportion of posterior ankle fracture blisters (*p* = 0.003).

**Table 4:** Fracture blister location

Location of fracture blisters on ankle	Number of blisters (%)	Post-hoc analysis *P* values*	Distribution *P* value†
Anterior	27 (23.1)	**1**	** < 0.001**
Posterior	14 (12.0)	**0.003**	
Medial	48 (41.0)	** < 0.001**	
Lateral	28 (23.9)	**1**	

*Exact binomial goodness-of-fit test, *P*-value corrected via Bonferroni correction method

†Exact multinomial goodness-of-fit test

## Discussion

While fracture blisters are a known complication of high energy pilon fractures, there is a paucity of data detailing their frequency, distribution, and influence on delaying definitive fixation. In this study, the average time to definitive surgery for pilon fracture patients with fracture blisters was found to be 14.2 days, nearly double that of patients without fracture blisters (7.9 days). In addition, patients with fracture blisters had a significantly higher proportion of 43C fracture patterns, while patients without fracture blisters had a higher proportion of 43B fracture patterns. Finally, 41% of fracture blisters were localized to the medial distal tibia and ankle, while only 12% occurred posteriorly. To the best of our knowledge, this study is the first to report on fracture blister location and frequency in pilon fractures.

The significant delay in definitive surgical fixation observed in patients with fracture blisters is consistent with prior literature [6, 10]. Fracture blisters are tense vesicles or bullae that arise secondary to a localized inflammatory response from soft tissue edema overlying a fracture site [4, 8, 12, 13]. While early fixation may reduce the incidence of fracture blisters [8], their presence is associated with poorer outcomes and complications, including infection and wound dehiscence [9, 14–16]. Therefore, current guidelines favor a staged approach with initial EF to provide stabilization and limit soft tissue insult, followed by staged ORIF once soft tissues are amenable [6, 17, 18]. Shah et al. found the average hospital stay for patients undergoing a staged approach to pilon fractures to be 13 days with 23% of patients being discharged after initial EF and prior to ORIF [19]. Identifying risk factors for delayed surgical fixation can not only aid in surgical planning, but also help identify candidates for discharge after initial EF with definitive ORIF scheduled at a later date. There is a scarcity of literature describing the length of delay in surgical care for patients with fracture blisters. Strauss et al. found a mean surgical delay of 6.8 days attributable to fracture blisters in pilon fracture patients; however, this data was largely limited by a low sample size of only four pilon fractures [10]. Our study, consisting of a cohort of 80 patients with fracture blisters, was consistent with Strauss et al. and found that pilon fractures associated with fracture blisters delayed time to ORIF by an additional 6.3 days. Considering this delay, patients with fracture blisters may be particularly good candidates for discharge after initial EF placement with an outpatient skin check prior to definitive fixation, as opposed to remaining in the hospital until definitive fixation.

In our study, the presence of fracture blisters was associated with higher energy type C fractures, which is consistent with prior literature [4]. Initial management of fracture blisters in these injuries is targeted towards controlling the early inflammatory phase and promoting re-epithelization by elevating the limb to reduce edema and swelling by increasing venous drainage [5, 20–22]. There is some evidence that cryotherapy can also be used to inhibit the inflammatory response by decreasing soft tissue temperature to control edema [23–25]. Strauss et al. found that treatment of fracture blisters with Silvadene improved re-epithelization and minimized soft tissue complications [10]. In a recent randomized controlled trial, Wiese et al. [26] demonstrated that silver-impregnated fibrous hydrocolloid dressings were a cost-effective alternative treatment option to Silvadene for fracture blister re-epithelialization.

Although previous studies have demonstrated that the aspirated fluid from fracture blisters are sterile [8, 27], Varela et al. [8] found that there was an increased occurrence of postoperative infections when the surgical incision crossed the blister site. Thus, the location of fracture blisters around the ankle may impact surgical approach and planning. Our study found an increased proportion of fracture blisters located over the medial ankle (41%, *p* < 0.001) and a decreased proportion over the posterior ankle (12%, *p* = 0.003). This may best be explained by the depth of the soft tissue envelope and distance of the skin from the underlying bone in each anatomic location. Previous studies have evaluated the efficacy of a posterolateral approach followed by a staged anterior approach in quality of fracture reduction and complication rate [28, 29]. However, none have evaluated the effectiveness of this two-staged procedure in the context of fracture blisters. Given the distribution of fracture blisters relative to the ankle, the staged posterolateral approach to the distal tibia is a promising technique that may reduce complication rates in patients with fracture blisters and should be a focus of future research.

In this study, no differences in rates of hypertension, smoking, alcohol use, peripheral vascular disease or diabetes were found between the fracture blister and control cohorts. However, prior studies suggest that comorbidities accelerate fracture blister formation due to baseline impairment of skin microvasculature [4, 5, 7]. One possible explanation for this discrepancy may be that the development of fracture blisters is significantly influenced by fracture severity while medical comorbidities play a more minor role in their formation. Additionally, as this study not quantify fracture blister severity, it is possible that comorbidities play a larger role in fracture blister severity and location. Further investigation evaluating the impact of these medical comorbidities in lower energy fracture patterns is warranted.

There are several limitations to our study. Firstly, given the limited number of AO/OTA 43A (extraarticular pilon) fracture patterns available for analysis, this study was likely underpowered for determining fracture blister prevalence in this subgroup. However, given the lower energy nature of extraarticular pilon fractures, it can be presumed that this fracture pattern would have lower rates of fracture blisters when compared to Type B or C fracture patterns. Secondly, three different surgeons operated on patients included in this study, allowing for minor variation in initial treatment. However, each participating surgeon followed a standardized protocol for the treatment of fracture blisters and contributed in providing care to both cohorts. Therefore, we believe this confounding effect is minimal and contributes to the generalizability of these results. Readiness for definitive ORIF was also a subjective assessment based on skin wrinkling and fracture blister re-epithelization, and may have had a confounding effect on data collected. Finally, we were unable to report on size or severity of fracture blisters. Further studies should include prospective, randomized controlled designs, and analysis of other fractures often susceptible to blisters, size of blisters, and efficacy of blister management techniques.

## Conclusion

Fracture blisters present a significant challenge for orthopaedic surgeons due to their association with postoperative wound complications and delays in ORIF. The presence of fracture blisters in pilon fractures are associated with significant delays in time to definitive fixation and higher energy fracture patterns. In this study we quantify the associated delay to definitive ORIF in patients with pilon fractures and associated fracture blisters. Additionally, we demonstrate that these blisters are associated with high energy fracture patterns and tend to develop over the medial aspect of the ankle, often sparing the posterior skin. Due to the substantial added delay in definitive fixation in the presence of fracture blisters, these patients may benefit from discharge after EF with outpatient skin and soft tissue evaluation several days after discharge prior to staged definitive treatment. Furthermore, given the location of blisters and timing of soft tissue readiness, one might consider acute EF application combined with ORIF via posterolateral approach followed by staged anterior fixation at a later date. Further research is warranted to assess outcomes using this approach. This investigation provides further insight into how to better plan and counsel patients on timing for definitive ORIF when fracture blisters are present.
